# A new human heart vessel identification, segmentation and 3D reconstruction mechanism

**DOI:** 10.1186/s13019-014-0161-1

**Published:** 2014-10-02

**Authors:** Aqeel Al-Surmi, Rahmita Wirza, Ramlan Mahmod, Fatimah Khalid, Mohd Zamrin Dimon

**Affiliations:** Department of Multimedia, Faculty of Computer Science and Information Technology, University Putra Malaysia, Selangor, Malaysia; Cardiothoracic Unit, Surgical Cluster, Faculty of Medicine, Universiti Teknologi MARA, Selangor, Malaysia

**Keywords:** 3D Model, Heart surgery, Image enhancement, Vessel segmentations

## Abstract

**Background:**

The identification and segmentation of inhomogeneous image regions is one of the most challenging issues nowadays. The surface vessels of the human heart are important for the surgeons to locate the region where to perform the surgery and to avoid surgical injuries. In addition, such identification, segmentation, and visualisation helps novice surgeons in the training phase of cardiac surgery.

**Methods:**

This article introduces a new mechanism for identifying the position of vessels leading to the performance of surgery by enhancement of the input image. In addition, develop a 3D vessel reconstruction out of a single-view of a real human heart colour image obtained during open-heart surgery.

**Results:**

Reduces the time required for locating the vessel region of interest (ROI). The vessel ROI must appear clearly for the surgeons. Furthermore, reduces the time required for training cardiac surgery of the novice surgeons. The 94.42% accuracy rate of the proposed vessel segmentation method using RGB colour space compares to other colour spaces.

**Conclusions:**

The advantage of this mechanism is to help the surgeons to perform surgery in less time, avoid surgical errors, and to reduce surgical effort. Moreover, the proposed technique can reconstruct the 3D vessel model from a single image to facilitate learning of the heart anatomy as well as training of cardiac surgery for the novice surgeons. Furthermore, extensive experiments have been conducted which reveal the superior performance of the proposed mechanism compared to the state of the art methods.

**Electronic supplementary material:**

The online version of this article (doi:10.1186/s13019-014-0161-1) contains supplementary material, which is available to authorized users.

## Background

One of the most issues of the extensive research activity over the past decades is the image homogeneous regions identification and image segmentation. Numerous researchers have been proposed different algorithms that elaborated the segmentation of greyscale images. However, the problem occurs in the colour image segmentation, which consists of much more information about the objects, especially medical images that are taken using modern acquisition devices.

Variation in the position, size and morphological pattern of vessels on the surface of the human heart and their main branches are important issues for performing and facilitating the surgery procedure, reduce surgical injuries, and enhancing the visual view of the human heart 3D model. Heart's vessels are categorized as arteries, veins, and capillaries. Human heart vessels have different characteristics. Arteries distribute the oxygenated blood to the human body, while veins return the deoxygenated blood to the heart and capillaries connect the veins to the arteries. This forms the blood circulation system.

**Figure 1:**
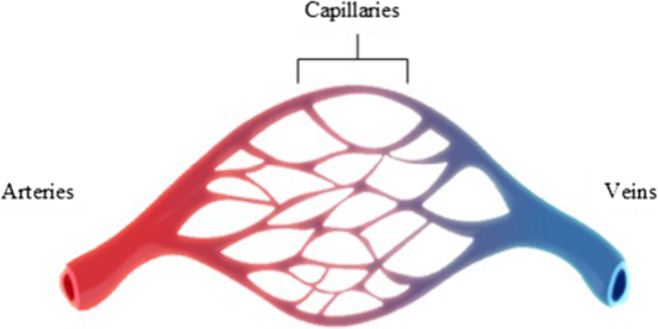
**Human blood vessels.**

Heart surface vessels have different colour appearance, which are darker than the other heart surface regions. The arteries are reddish due carrying oxygenated blood while the veins are bluish in colour as they carry deoxygenated blood as shown in Figure 1.

**Figure 2:**
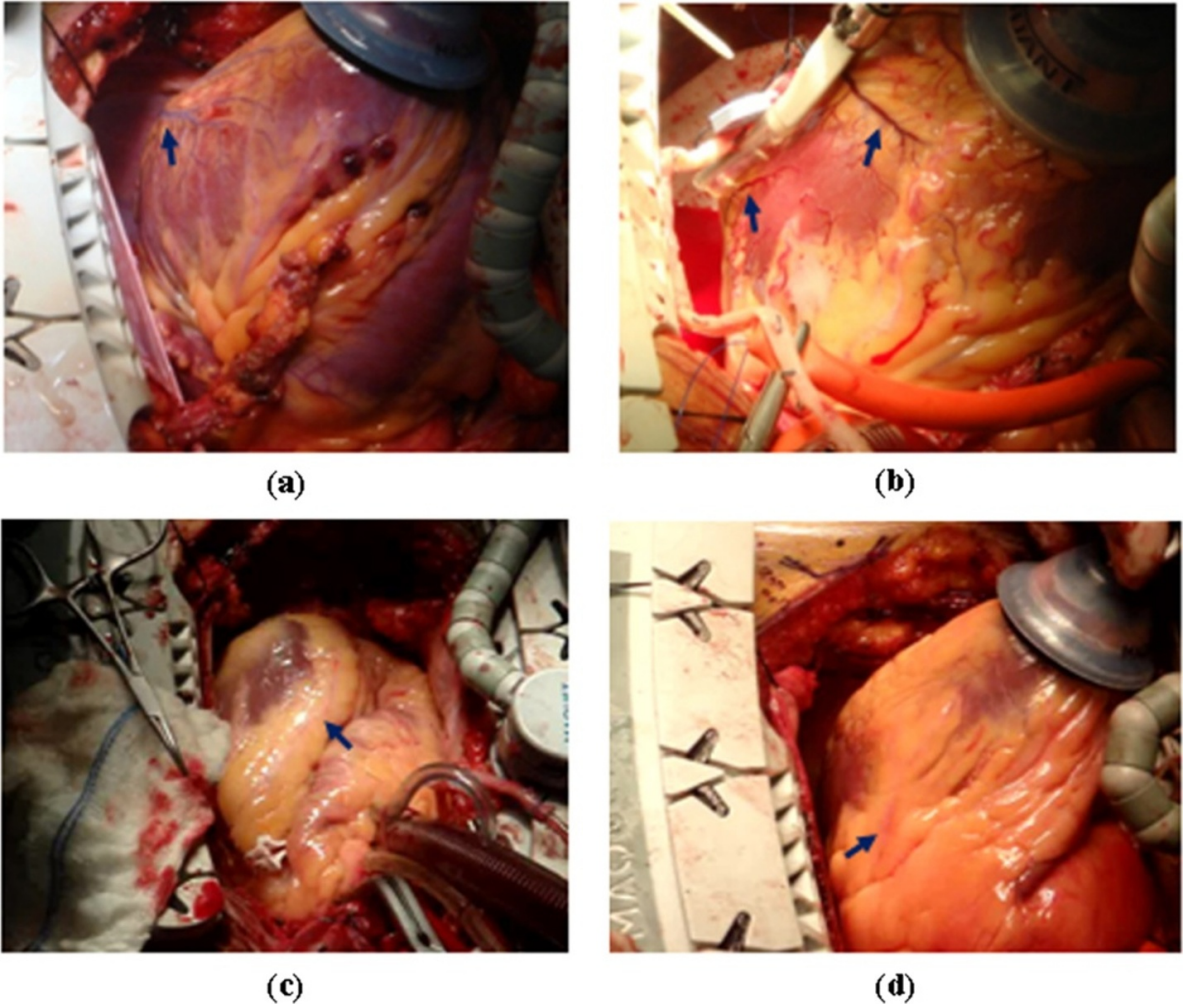
**Different views of the human heart surface vessels. (a)** Deoxygenated blood vessel. **(b)** Oxygenated blood vessel. **(c)** Bloodless vessel ROI. **(d)** Fats cover the blood vessel ROI.

Furthermore, fats usually cover the surface of the human heart, which also covers some of the surface vessels. However, when the heart is pulled up by Vacuum Positioner System [1], the vessels appear clearly on the other side of the heart surface as in Figure 2a and b. Usually the vessels are embossed over the surface of the heart. On the other hand, the vessels ROI that the surgeons are looking for cannot be recognized easily and it appears whitish due to its bloodless and the fats that covered them, as shown in Figure 2c and d.

The vessel segmentations of the human internal organs have a long standing in computer graphics, especially images from medical imaging devices, Magnetic resonance imaging (MRI) or Computed Tomography scan (CT), such as retina or coronary artery images.

Recently, the new medical imaging devices such Computed Tomography Angiogram (CTA), endoscopic, and digital cameras produce colour images. These images can be saved with different colour space extensions. The RGB model is the most common extension used in recent image acquisition devices. According to the Tri-stimulus theory [2] three components can represent the colour resulting from three separate colour filters S_A_, A = R, G, B, for light radiance E(λ) according to the following equation:

1 $$\begin{array}{l} R=\int_{\lambda }E\left(\lambda \right)S_{R}\left(\lambda \right)d\left(\lambda \right)\\ G=\int_{\lambda }E\left(\lambda \right)S_{G}\left(\lambda \right)d\left(\lambda \right)\\ B=\int_{\lambda }E\left(\lambda \right)S_{B}\left(\lambda \right)d\left(\lambda \right) \end{array}$$

Many colour spaces are based on human colour perception, such as Hue, Saturation, and Intensity (HSI) components. Other colour spaces follow the Munsell colour system [2]. For example, HSI can be transformed from RGB using the following equations:

2 $$I=\frac{R+G+B}{3}$$

3 $$S=1-\left(\frac{3}{R+G+B}\right)*\mathrm{minRGB}$$

Where minRGB is the minimum of R, G, and B.

4 $$H=\mathrm{co}s^{-1}\frac{\left(0.5*\left(R-G\right)+\left(R-B\right)\right)}{\sqrt{{\left(R-G\right)}^{2}+\left(R-B\right)\left(G-B\right)}}$$

In addition, another colour space tested in the current experiment for vessel segmentation is Hue, Saturation, and Value (HSV). The conversions given in [3] by Travis assumes normalised RGB values before conversion using the following equations:

5 Delta=maxRGB-minRGB

where maxRGB is the maximum of R, G, and B.

6 V=maxRGB

7 $$S=\frac{\mathrm{Delta}}{\mathrm{maxRGB}}$$

8 $$H=\left\{\begin{array}{ll} \frac{G-B}{\mathrm{Delta}}*60 & R=\mathrm{maxRGB},\mathrm{Delta}\neq 0\\ 2+\frac{B-R}{\mathrm{Delta}}*60 & G=\mathrm{maxRGB},\mathrm{Delta}\neq 0\\ 4+\frac{R-G}{\mathrm{Delta}}*60 & B=\mathrm{maxRGB},\mathrm{Delta}\neq 0\\ 0 & \mathrm{Delta}=0 \end{array}\right.$$

Digital coding international standard YCbCr colour space was also tested for the segmentation of the heart surface vessels as follows:

9 Y=0.299*R+0.587*G+0.114*B+16

10 Cb=-0.169*R-0.331*G+0.500*B+128

11 Cr=-0.500*R-0.419*G-0.081*B+128

Finally, the CIELab colour space was used in the vessel segmentation experiment. The equations will not be mentioned here due to the need to transform to CIEXYZ first, then to CIELab. For more information please refer to Ford and Roberts [4].

## Related work

Several studies have generally investigated vessel segmentation algorithms, such as region growing [5],[6] which starts by choosing a seed pixel. Then, the surrounding pixels are added based on their properties and similarities such as texture, shape and intensity. This results in a segmented region within the image. However, the seed point selection can produce different segmentation results when different seed points are selected. In addition, thresholding segmentation methods [7],[8] present a simple process and require less computational time. However, to obtain correctly segmented regions it is required to use more than a single threshold to segment the vessels due to the characteristics of the vessels with different intensities, which is time consuming to find optimal threshold values.

Numerous researchers [9],[10] applied clustering, which is used for segmentation based on analysing the input images by dividing into different clusters, such as the popular K means clustering method. These methods are quite fast compared to other segmentation methods. However, the k value determination can be problematic as an unsuitable choice can give a poor segmentation result. Other researchers use Watershed segmentation methods [11],[12] as a region-based process. However, it is applied to the image gradient rather than to the image directly. These methods can work well for an image that has homogeneous regions in a simple biomedical image. On the other hand, complex medical images, such as images from medical imaging devices usually contain many inhomogeneous regions.

Moreover, the vessel pixels have different intensities in addition to the noise, which appears as pixels with low intensities, which causes previous general segmentation algorithms to fail to segment vessels correctly. Vessels in a human body have various forms from one organ to another. Several methods are available to investigate vessel segmentation, especially from the retina, brain or heart vessels. Data input to those methods are even colour or greyscale images. These images begin with transformation to a greyscale and then removing the background in addition to other pre-processing steps in order to perform the vessel segmentation.

Several studies have been published on the vessel segmentations. Reviews of vessel like structure in CTA images was proposed, whereas several vessel segmentation methods were introduced for the vessels in the human leg [13]. Review research done by Suri et al. [14],[15] begins by spotlighting the pre-processing filtering of Magnetic Resonance Angiogram (MRA) to remove the image background. This is followed by an overview of the vessel segmentation algorithms in which Suri divided the vessel segmentation into two groups, classified as non-skeleton and skeleton. Non-skeleton based approaches directly compute the vessels, while skeleton-based approaches use cross-sections to reconstruct the vessel lumen after vessel skeleton computing.

Quantification problems of vessel analysis were investigated by Buhler et al. [16], such as boundary detection, centreline extraction and projection techniques. Furthermore, Kirbas and Quek [17] divided the vessel extraction algorithms into six approaches, namely model based, neural network based, tracking based, artificial intelligence based, pattern recognition, and tube-like object detection approaches. In recent method for vessel segmentation, Lesage et al. [18] assumed a model based on the geometry and appearance of the vessels and image feature extraction to perform vessel segmentation.

On the other hand, to our knowledge, no works have dealt with real and colour human heart input images for segmentation of vessels since most of the current methods input dataset type are from medical imaging devices, such as CTA or angiogram images. In contrast, the proposed mechanism employs real input data to be used in order to know the vessel ROI that will be operated on. From here, we can see the important of dealing with real human heart images to identify where to perform the surgery without surgical injuries and reconstruct the 3D vessel model.

The vessel segmentation process is necessary in order to identify the ROI, then the 3D reconstruction process is used to visualise segmented vessels. This article explains the methods and the process as well as present several experiments that have been conducted for heart surface vessel segmentation. A semi-automatic mechanism is applied based on a seed point segmentation algorithm by using a hybrid mechanism to identify the vessel ROI. Finally, a Bezier curve algorithm is applied to smooth the reconstructed 3D model based on the segmentation results.

## Methods

Vessel segmentation aims to highlight the blood vessels in images of the surface of the human heart to allow the surgeon to determine the ROI of the vessels so that correctly perform cardiac surgery. Another aim is to smooth the reconstructed 3D vessels model. The data used in this research are real human heart colour images. The data was collected during open-heart surgery (cardiac surgery) under supervision of specialist cardiac surgeons. Moreover, the data for each patient was taken using Sony Cyber-Shot DSC-T30 Digital Camera, with a focal length between 38-114 mm, resolution 7.2 megapixels, and 3x optical zoom with aperture range f3.5-f10. In addition, the data saved in a standard JPG format for each image in a single folder for each patient. The patients were from different genders male and female with average age 56 years old. The study was approved by the ethics committee of the Universiti Putra Malaysia (UPM) and Universiti Kebangsaan Malaysia Medical Centre (UKMMC).

**Figure 3:**
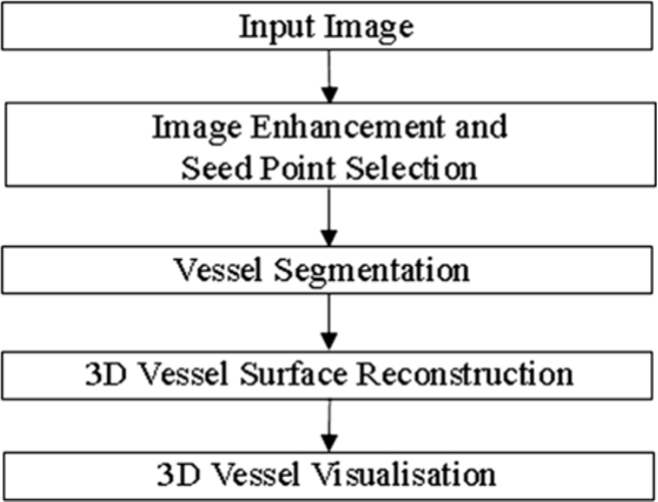
**Vessel segmentation and 3D vessel reconstruction framework.**

Three main steps was used for the proposed methodology for the 3D vessel segmentation and reconstruction algorithm. First, segmentation process of the heart vessels. Second, the 3D heart vessel reconstruction process. Finally, curve fitting process and visualizing the result in R3 space. The following subsections are dedicated to explain the proposed methodology in detail. The overall framework of this algorithm is illustrated in Figure 3.

**Figure 4:**
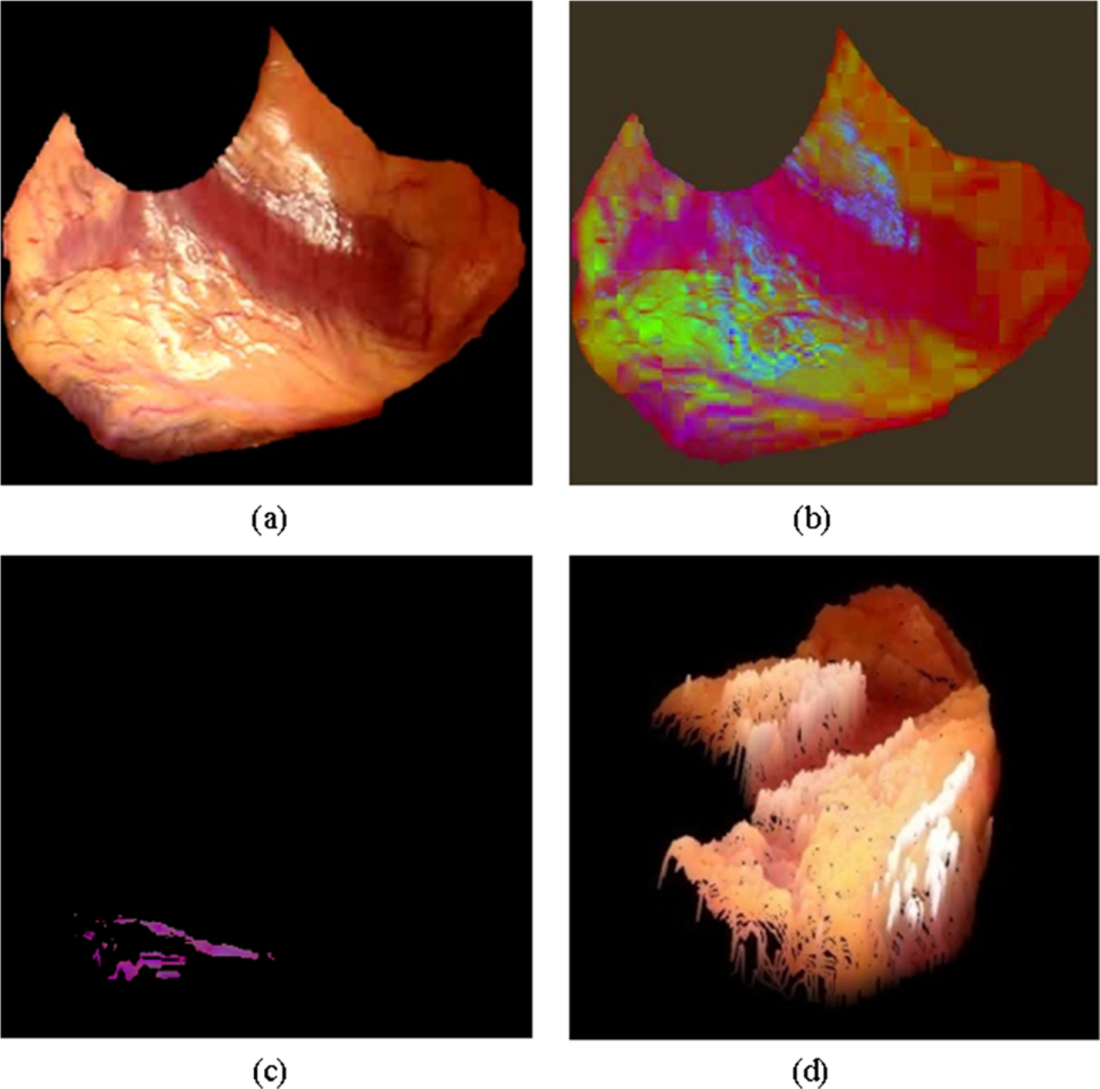
**Vessels segmented by proposed segmentation mechanism and visualisation over the 3DHHM surface model. (a)** Original human heart image. **(b)** Decorrelation Stretch process. **(c)** Segmented vessel ROI. **(d)** 3D vessel visualisation (white).

The input to the proposed method is a colour human heart image that has vessels on its surface as shown in Figure 4a. The enhancement process applied to the input image and then select the seed point from the vessels region for the vessel segmentation process as illustrated in Figure 4b. Moreover, Figure 4c show the output of the segmentation process of the vessel ROI, which used as the input for the 3D vessel reconstruction process. Finally, curve-fitting process applied to the segmented vessel ROI, and then visualizing the 3D of the vessels over the 3D heart model in R3 space as shown in Figure 4d.

### Heart surface vessel segmentation

The vessels of the surface of the human heart vary in perception. The blood full vessels with reddish or bluish surface colours does not require surgical intervention. However, bloodless vessels with whitish surface colour are significant to the surgeons since it indicates as a vessel ROI where the surgery will be performed. These vessels with whitish surface colours are difficult to segment using current segmentation methods due to their being bloodless, covered by fat, and are affected by lighting reflections on the surface of the heart as its wet surface. The reflections affect the perception of the vessels. The reflection can be removed be the method proposed in [19]. Moreover, the proposed segmentation method initially enhances the original image of the human heart using a Decorrelation Stretch [20] as shown in Figure 4b.

The Decorrelation stretch has found increasing usage over the past decade, especially in satellite images, such as images taken from a long distance or image features that are not easy to identify. The Decorrelation stretch used to enhance the image colour separation by maximizing the difference between different combinations of the three bands of RGB colour channels. Several experiments conducted using image processing techniques with different colour spaces to overcome the difficulties of the segmentation methods of the human heart surface vessel since it bloodless and effect by other factors, however, the results were not promising. In the other hand, an image enhancement was applied to differentiating between surface colours to identify the vessels ROI and it obtained a better result. To our knowledge, the use of the Decorrelation stretch technique is proposed for the first time to enhance the real human heart image.

Furthermore, the semi-automatic mechanism is proposed for the vessel segmentation process based on a single seed point. In this method user interaction is required to choose a single seed point from the region of the heart surface vessels. The seed point is proposed for the reason that the surface vessels do not appear in all human heart images due to the fats, which covers the surface of the heart as mentioned earlier. This means that the vessel segmentation will not run for the all input images, except for the nominated images with vessels on its surface. Moreover, once the user clicks on the heart image it implies that this current image has surface vessels, and this single click assigned to be the seed point to segment the vessels in the ROI of that image.

In addition, the pixel clicked by the user will be analysed to determine its colour information in the RGB channels, with each channel value taken separately. This pixel value considered as a threshold value, however, the segmentation process will detect only the pixels with same value while the vessel regions have different pixel values. For this, a constant value is added to all channel values as a range for the sake of segmenting the vessel ROI, this value is experimentally found. If it is within the range, then it is segmented as vessels within the ROI. Although the seed point has some limitations, conducted experiments found that it is the best technique to run on real human heart images due to its simplicity and effectiveness segmentation technique for the input data image type. In addition, several experiments have been conducted using a number of segmentation methods beside testing those methods with different colour spaces.

### 3D vessel algorithm

This section gives attention to the reconstruction of a 3D vessel model. Several methods have been investigated 3D vessel reconstructions from multiple images. To our knowledge, none has discussed reconstruction of a 3D vessel model from a single-view image of the real human heart colour images. The proposed algorithm starts with the extraction process of the pixel information positions and intensity of the vessels, whereas the pixel positions represent x and y-axes, while pixel intensity represent the z-axis to build the 3D model. In addition, the colour information of the pixels of the vessels.

The vessels data extracted from a single heart image were saved to a data file as a cloud of points, each point has three axes x, y, and z in the matrix form for each axis. This means the x_i_ = [x_1_, x_2_, ......, x_n_], y_i_ = [y_1_, y_2_, ......, y_n_], and z_i_ = [z_1_, z_2_, ......, z_n_], where *n* is the number of points in the data file. According to the computer graphics, 3D projections and transformation principles the matrix of 3D data would be projected into R3 space. Moreover, the 3D reconstruction algorithm generated a 3D data file automatically from the colour heart image by getting the information of each pixel values and colours. Upon completion they were saved as text files (x, y, and z) and (R, G, and B), respectively.

In addition, the cloud of points projected in the R3 space used the OpenGL library as Volumetric Picture Element (voxel) which can be zoomed, rotated and transformed that showed the model as coherent real vessels of the human heart surface. According to the information held in the data file the projection would register the voxel positions in their specified locations according to the corresponding location from the heart surface vessels. Moreover, those voxel's positions were in three Cartesian planes, xyz-axes, in which those planes were orthogonal to each other in the 3D space. Furthermore, each voxel had its own colour as the original heart vessel image that made the model as real as a real human heart surface vessel.

The nature of the vessels is embossed on the heart surface with maximum height equal to 2.5 mm. To rise up the segmented vessels, the maximum height value are added to the intensity value, which represent the z-axis, for each extracted pixel in the vessels ROI. In addition, only the main vessel branches are required to be segmented, since the smallest branches not require any surgical intervention as stipulated by the expert surgeons.

### Vessel curve fitting and 3D visualisation

The use of pixel intensity value as z-axis has great effect to generate a 3D model. However, it has a tiny drawback to the final result, especially when enlarging the 3D vessel model, the smoothness of the 3D result model is reduced and appears as sharp edges on some regions of the 3D model's surface that needs a smoothing process to the region. Several numerical methods in computer graphics can solve the surface smoothness problem using curve and surface interpolation or approximation techniques. Interpolation techniques have several drawbacks such as it passes through all data points. In contrast, approximation techniques do not pass through all data points while still captures the surface shape. Therefore, approximation techniques was used to smooth the 3D vessel surface model due to its desirable features.

Bezier curves and B-splines are two of the popular models for numerical analysis and widely used in computer graphics and computer-aided design. These curves have a good geometric property in that if changing one of the points we change only one portion of the fitted curve, a local effect. Compare to the cubic splines, changing only one point might have a global effect [21]. In addition, they do not ordinarily pass through the all given data points. Bezier curves is used frequently to smooth a model surface curve. B-spline is considered as a special case of Bezier curve. However, it requires more information, such as the degree of the curve and a knot vector. In this manner, the Bezier curve approximation technique will be used to ensure the smoothness of the 3D vessel of the human heart surface. This is followed by projecting the 3D vessels into the R3 space after fusing it together with the 3DHHM.

## Implementation

This section gives attention to prove the workability of the human heart surface vessel segmentation and 3D reconstruction algorithms. The implemented algorithm begins with the segmentation process to identify the vessel ROI, then reconstruct and visualise it in R3 space. The algorithm is implemented systematically according to the proposed methodology. A Graphical User Interface (GUI) has been created to implement the algorithms, test the experiments, and present the results. The GUI was created using Microsoft Visual Basic 6 (VB6). Moreover, different programming languages have been implemented for the 3D vessel reconstruction algorithm, visualisation, and their executable files called by the VB6 program.

The reconstruction of the 3D vessels of the surface of the human heart starts with a seed point selected by the user. The seed point is assumed to be from any position within the vessel region. The range of the vessel detection process to identify the vessel region is extended by adding a constant value to the seed point. For example, if the chosen seed point value is equal to 125, then a constant value is added, for instance 20, so the vessel detection range will be between 105 and 145. After the detection process of the vessel regions, the next processes takes place to extract the pixel information (x, y, and z) and (R, G, and B) and then this information is saved into data and colour files, respectively. Next, a Bezier curve is used to interpolate the cloud of points for the final 3D reconstructed result. Finally, the process of deriving the 3D reconstruction model from the segmented vessels is accomplished by a VC++ program with the OpenGL library to visualise a smooth 3D model of the vessels of the human heart.

**Figure 5:**
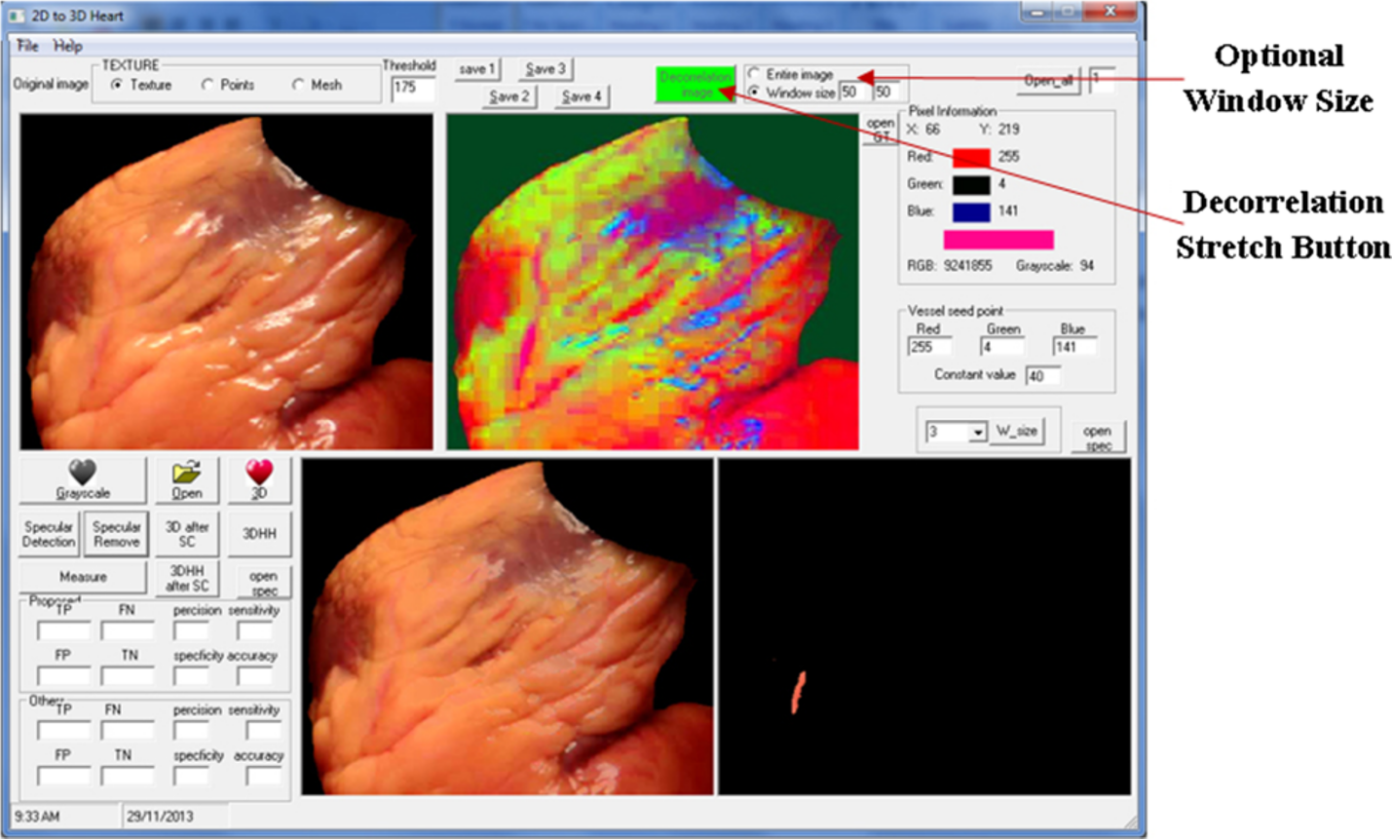
**GUI design for vessel segmentation and 3D reconstruction processes.**

A snapshot of the implemented GUI for the vessel segmentation and reconstruction processes is shown in Figure 5. The interface allows a user to proceed with the original colour image when the vessels are clearly notable. Otherwise, it enhances the original image using the Decorrelation stretch by pressing the green button in the interface.

The user may optionally specify a rectangular subarea for vessel identification, which permits a larger variety of colours within the ROI instead of using the entire image. Using the entire image would be time consuming and the image would need further processing to remove the unwanted regions. Moreover, the proposed subarea allows the algorithm to be fast and produce accurate ROI identification to simplify the view of the vessel ROI for the surgeons. The proposed subarea size begin assumed is 50×50 pixels around the selected seed point. For the segmentation algorithm, the user is required to click on the image of the heart that includes vessels on the surface. Then the vessels will be automatically segmented and viewed as shown in Figure 5 (bottom right corner image in the GUI).

**Figure 6:**
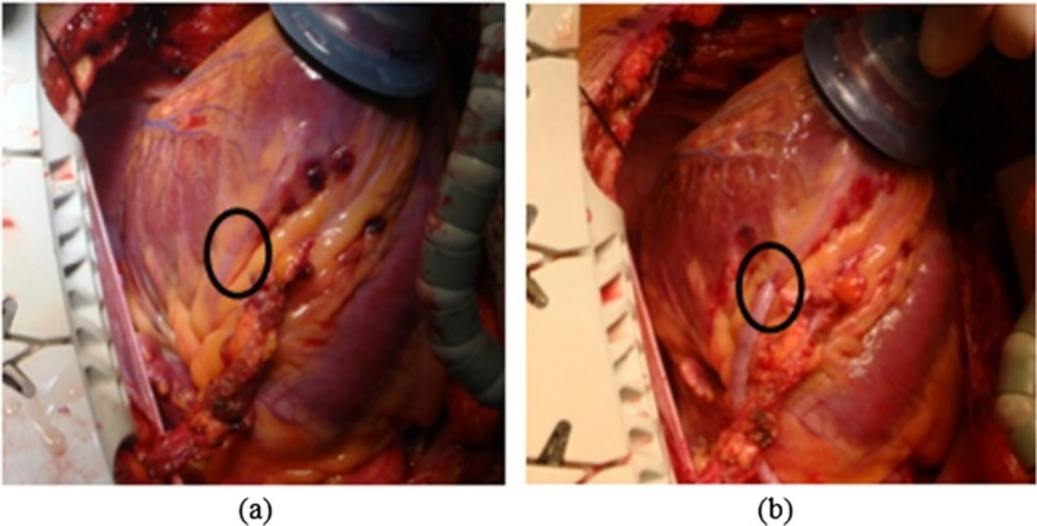
**Human heart image. (a)** Before cardiac surgery. **(b)** After cardiac surgery in the vessel ROI.

Furthermore, the vessel ROI where the surgery is to be performed does not encompass all the vessels of the heart surface as the surgeons operate on only a small region. Figure 6 illustrates the surface images of the human heart before and after surgery is done within the vessel ROI.

**Figure 7:**
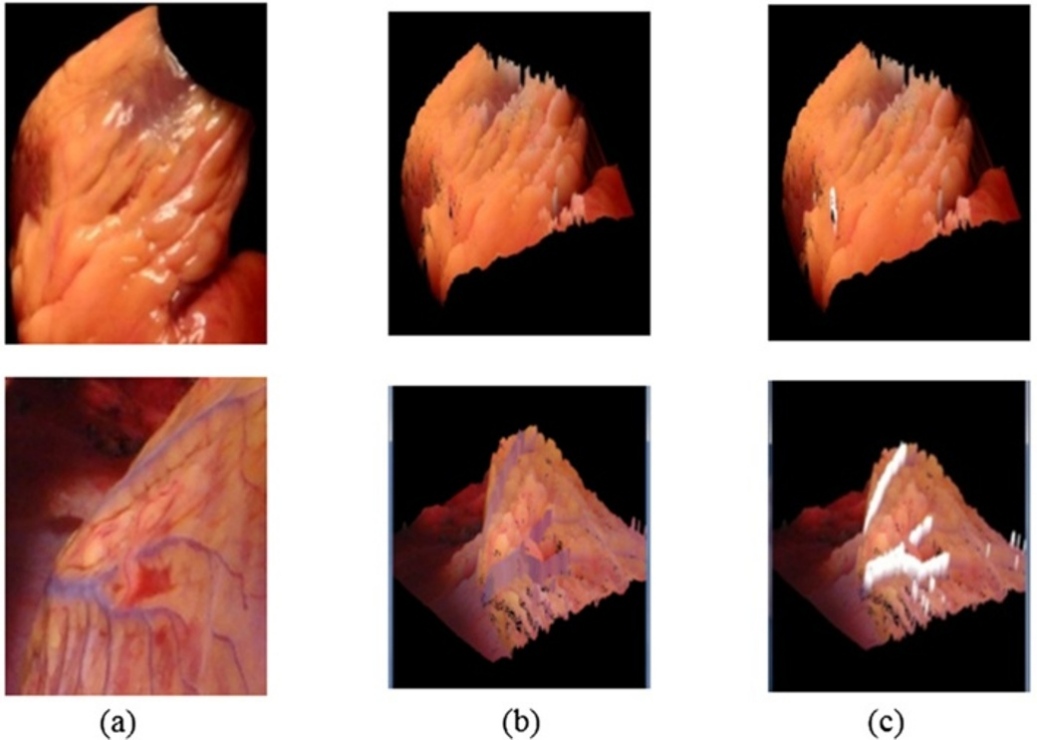
**Result of the proposed 3D vessels fused with the 3DHHM surface model. (a)** Original human heart images. **(b)** 3DHHM with original vessel colour. **(c)** 3DHHM with white vessel colour (demonstration only).

Finally, the 3D reconstruction algorithm is executed to return a 3D model of the human heart surface fused with the 3D vessel model projected in the R3 space. Figure 7 shows the results of a 3D human heart surface model with reconstructed 3D vessels.

The results illustrate the 3D vessels being fused with the 3DHHM model as shown in Figure 7. The 3D vessels in white in the colour Figure 7c is for the sake of demonstration only.

## Results and discussion

The 3DHHM results show the surface of the whole heart as one surface, i.e. without 3D vessels on the heart surface even if they clearly appear. This is because the vessel regions have a darker colour compared to the other regions of the heart surface. Most of the laser scanner devices, 3D reconstruction methods, and medical imaging devices ignore and do not consider the dark regions of the object under examination with the result that there are holes instead of raised vessels. In contrast, the proposed mechanism is able to reconstruct the vessels by developing a 3D vessel reconstruction mechanism using segmentation process and then 3D reconstruction and visualisation process.

The experiments in the segmentation of the surface vessels of the human heart in this section are categorised into three main parts, namely segmenting using different colour spaces, different medical image segmentation methods, and different other segmentation methods. Each of these categories are experimentally tested and evaluated.

### Comparison with respect to different types of colour spaces

Image segmentation objective is to partition a given input image into different regions. Colour segmentation approaches are based on monochrome segmentation approaches operating in different colour spaces. Humans perceive colour as a combination of Tri-stimulus R, G, and B, usually called primary colours. From R, G, B representation, we can derive other kinds of colour spaces by using either linear or nonlinear transformations as mention earlier. There are several studies trying to identify which is the best colour space to represent the colour information, but there is not a standard about which is the best choice. However, some researchers identify the best colour space for a specific task.

**Figure 8:**
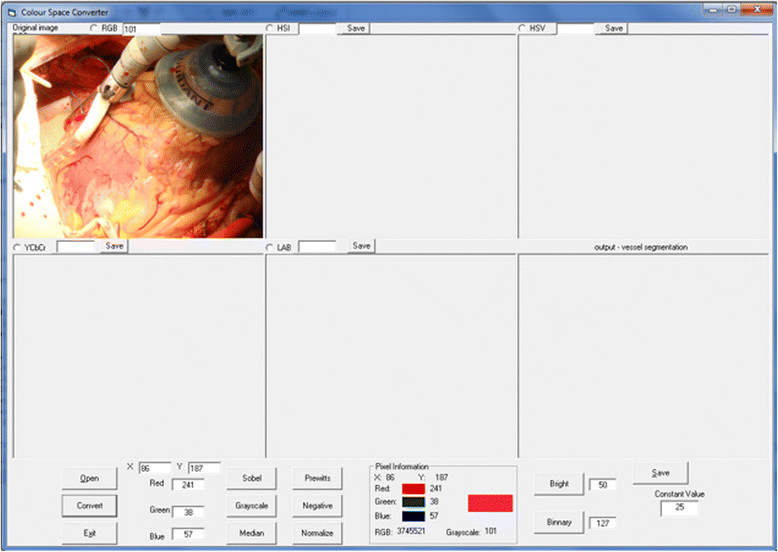
**The GUI design for colour space transformation.**

Colour space conversion algorithms were implemented systematically in the experiment according to the above mentioned equations and a new GUI interface created using VB6 was developed for the segmentation process of the human heart surface vessels using the different colour spaces. Moreover, different filters and edge detection techniques were implemented for the same segmentation purpose. The segmentation GUI interface is shown in Figure 8.

**Figure 9:**
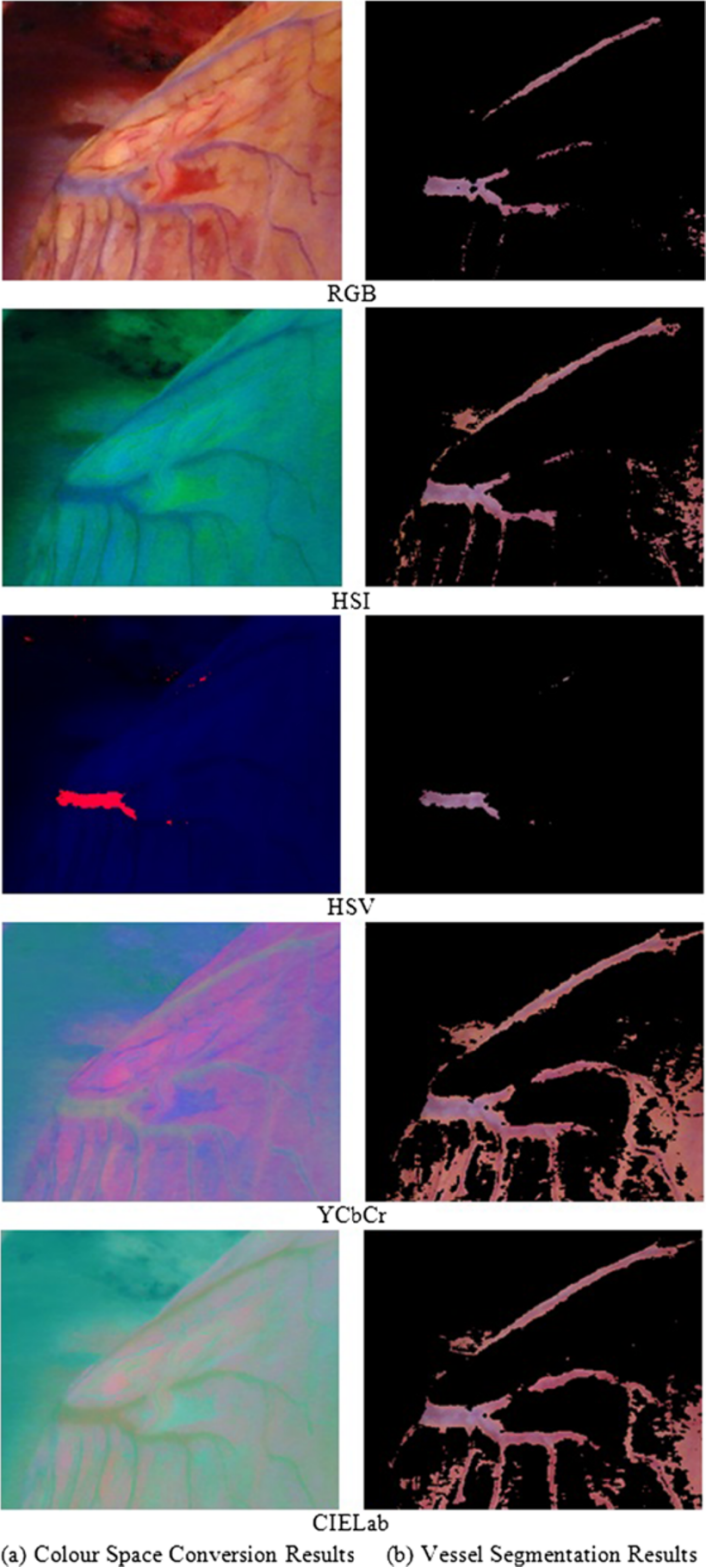
**Results of vessel segmentation using different colour spaces.** **(a)** Colour space conversion results. **(b)** Vessel segmentation results.

The techniques and algorithms are implemented and tested to prove that the RGB colour space segmentation performance is improved and that it produces accurate results compared to the other colour spaces. Moreover, it is time consuming for the conversion process to another colour space in addition to which colour space to transform. Furthermore, most of the modern acquisition devices use the RGB model. For these reasons, the proposed methodology works directly with RGB colour space images taken by a digital camera in order to save time and produce an accurate result, as shown in Figure 9.

In addition, Figure 9 shows the results of the conversions from different colour spaces and the experimental results of each of colour spaces conversion process of the vessel segmentation algorithms. This visually output proves that better result can be obtained using the RGB colour space.

The proposed algorithms had been implemented and tested. Quantitative method were applied to determine the accuracy performance metric [22] of the RGB colour space compare to other colour spaces. The quantitative tests for this dataset given by the performance metric from Table 1.

12 $$\mathrm{Accuracy}=\frac{\mathrm{TP}+\mathrm{TN}}{\mathrm{TP}+\mathrm{TN}+\mathrm{FP}+\mathrm{FN}}$$

**Table 1:** **A confusion matrix for positive and negative tuples**

Actual\predicted	Segmented vessels correctly: yes	Segmented vessels correctly: no
**Segmented vessels correctly: yes**	True Positive (TP)	False Negative (FN)
**Segmented vessels correctly: no**	False Positive (FP)	True Negative (TN)

**Figure 10:**
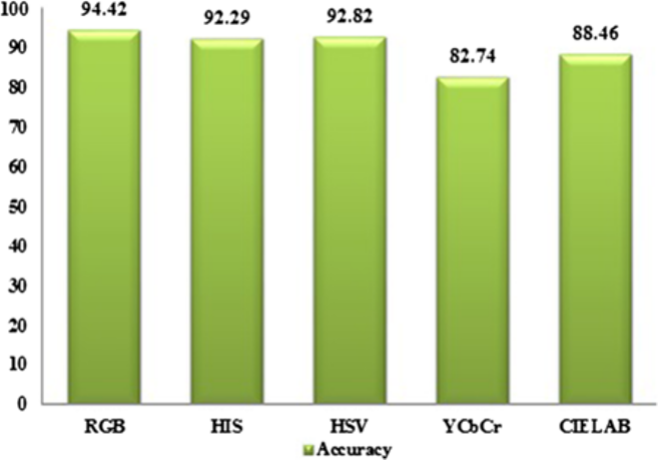
**Accuracy results for different colour spaces.**

The evaluation started by manually creating a set of ground truths by labelling the vessel pixels in a set of the human heart images from different male and female patients with an average age of 56 years old. Then, the performance of the proposed method was compared with the other colour spaces. Figure 10 shows the accuracy of vessel segmentations using different colour spaces compared to the ground truth.

### Comparison with respect to vessel segmentation methods of medical images

Medical imaging devices produce images of the internal human body organs, such as MRI, CT, Ultrasound, and Echocardiography. These devices produce video or still images, and then a frame grabber is used to capture the video signal for further processing and operations. Moreover, the results are greyscale images of the internal human organs under examination. Several techniques and methods are proposed for further video or image processing to segment specific information (such as tumours, vessels, arteries) in order to be used for educations, medical science or clinical purposes. In contrast, colour images that are acquired by new devices, such as endoscopic images, digital cameras, or other digital devices. However, it suffer from the segmentation process of the ROI due to the colouring of the image and the pixel similarities. This results in incorrect segmentation when applied to medical imaging segmentation methods. Hence, it draws attention to the experiments of vessel segmentation of a single-image of the human heart taken by a digital camera.

Several studies have been published on vessel segmentation [23]-[27]. These studies focus on segmenting vessels from retina or heart images. However, extensive processing steps are required to initially convert the images to greyscale, followed by several pre-processing, such as filtering, background and artefact removal. Moreover, different segmentation methods have been proposed with quantitative and qualitative segmentation results [28],[29]. However, the objective comparison technique used in a few of these methods to compare the substantial datasets, and the private databases used in each of the published mechanisms for experiments with a different reference contour description, result in difficulties when performing objective comparisons between these methods.

**Figure 11:**
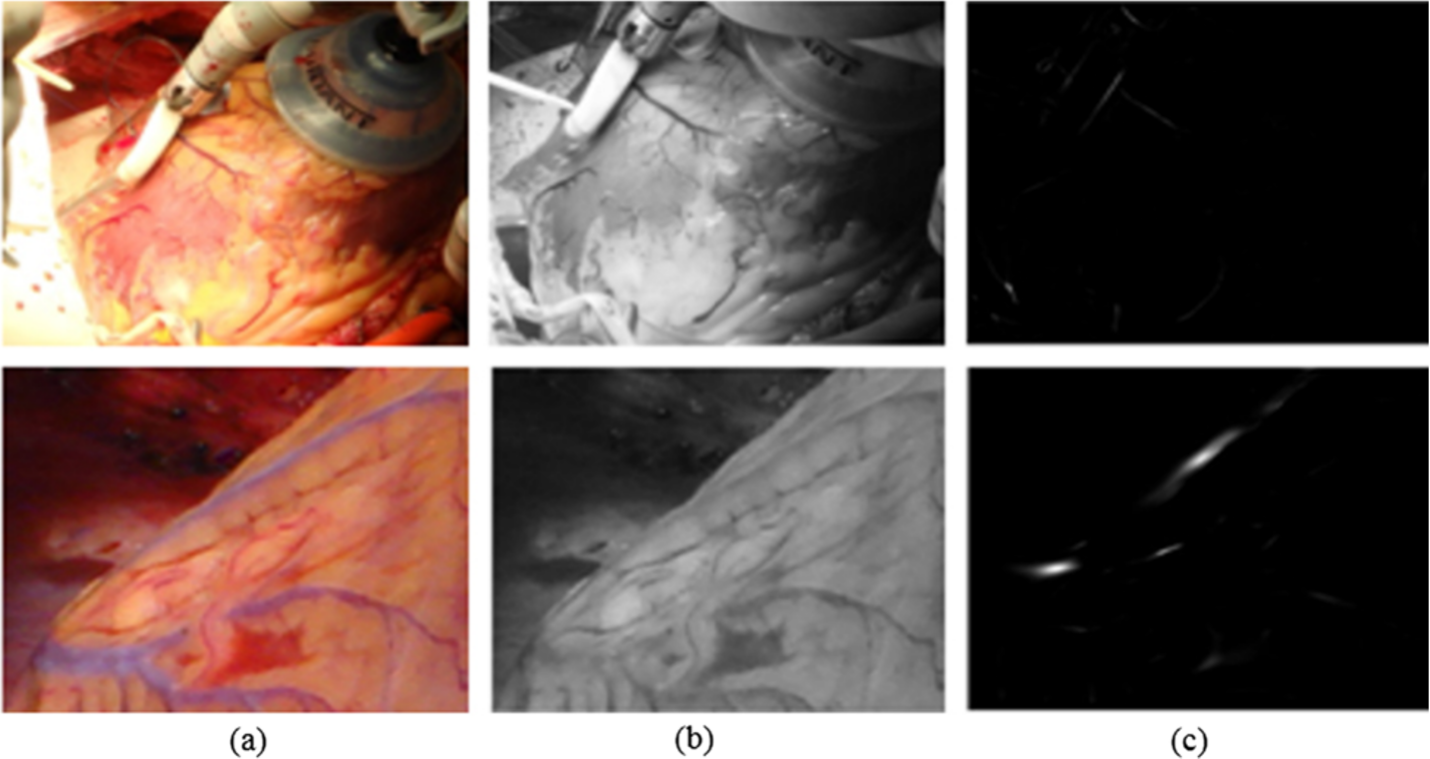
**Human heart vessel segmentation result using Frangi algorithm**[28]**. (a)** Original human heart images. **(b)** Greyscale heart images. **(c)** Result of Frangi algorithm.

The algorithm proposed by Frangi [28] is used to test and evaluate images of a real human heart. Figure 11 shows the results of the Frangi algorithm. Frangi starts by converting a colour image into a greyscale image as in Figure 11 (b), followed by the vessel segmentation process, as shown in Figure 11 (c).

**Figure 12:**
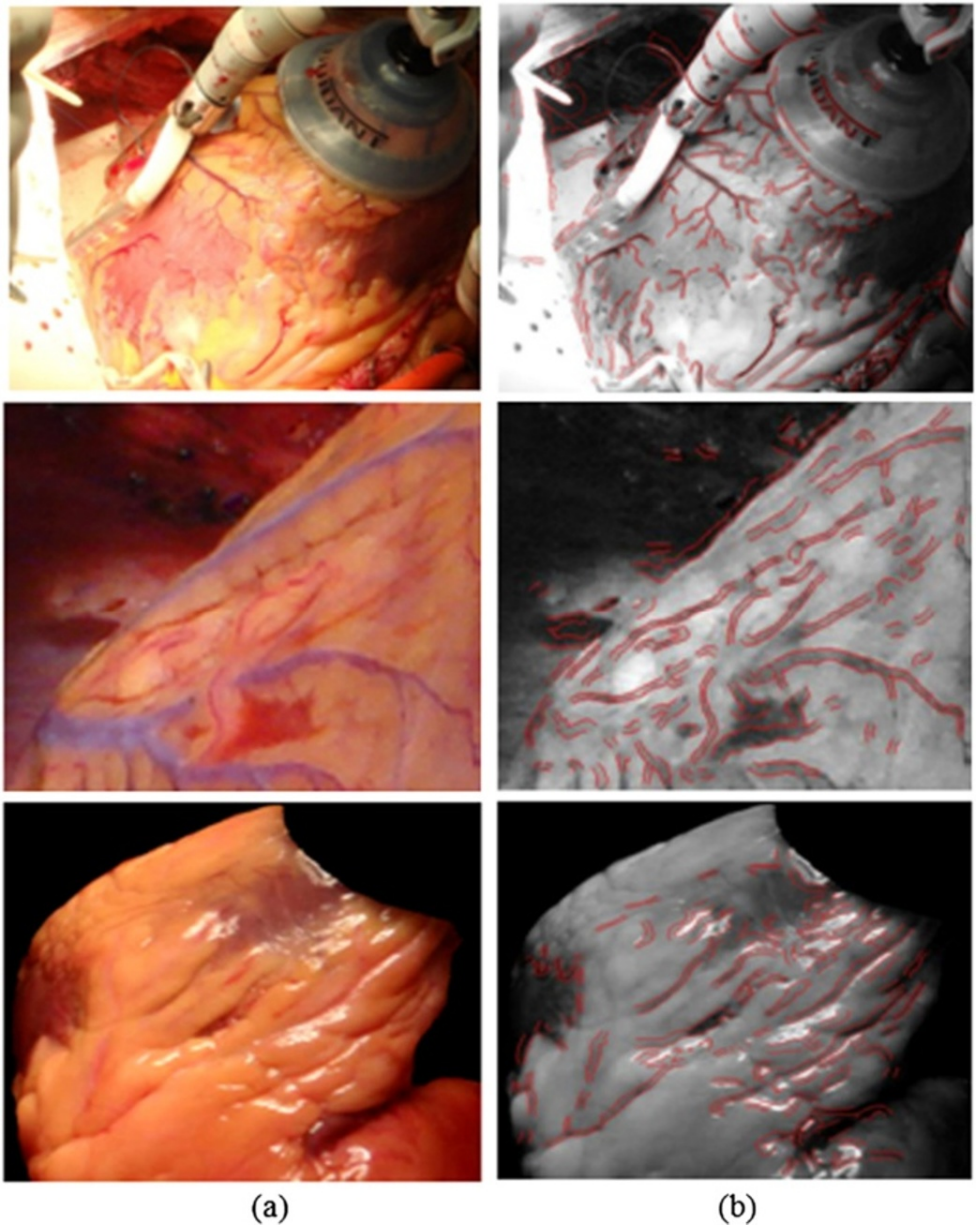
**Human heart vessel segmentation result using ARIA algorithm** **[**[30]**]** **. (a)** Original human heart images. **(b)** Results of ARIA algorithm.

Retina algorithms were also examined and tested using images of a real human heart. The results of the ARIA method proposed by Bankhead et al. [30] are shown in Figure 12. Moreover, Bankhead developed an excellent algorithm for retina vessel segmentation, which has produced promising results. However, when applied to images of a real human heart the result obtained not only includes the heart surface vessels, but also wrong vessels segmented regions.

According to the experiments and results from Figures 11 and 12, medical imaging segmentation techniques cannot produce correct results when applied to images of a real human heart.

### Comparison with respect to other segmentation methods

Vessel segmentation of the surface of the heart is crucial for cardiac surgical and 3D reconstruction. To our knowledge, no studies available that have investigated segmentation from colour images of the surface vessels of a real human heart. Several general methods have been used for segmentation of medical images. As well as, hybrid techniques are often used for solving different segmentation problems. Existing segmentation methods can be divided into several categories, such as thresholding, region growing, watershed, and classifier methods. In addition, other methods are not mentioned here due to being unrelated to heart vessel segmentation.

**Figure 13:**
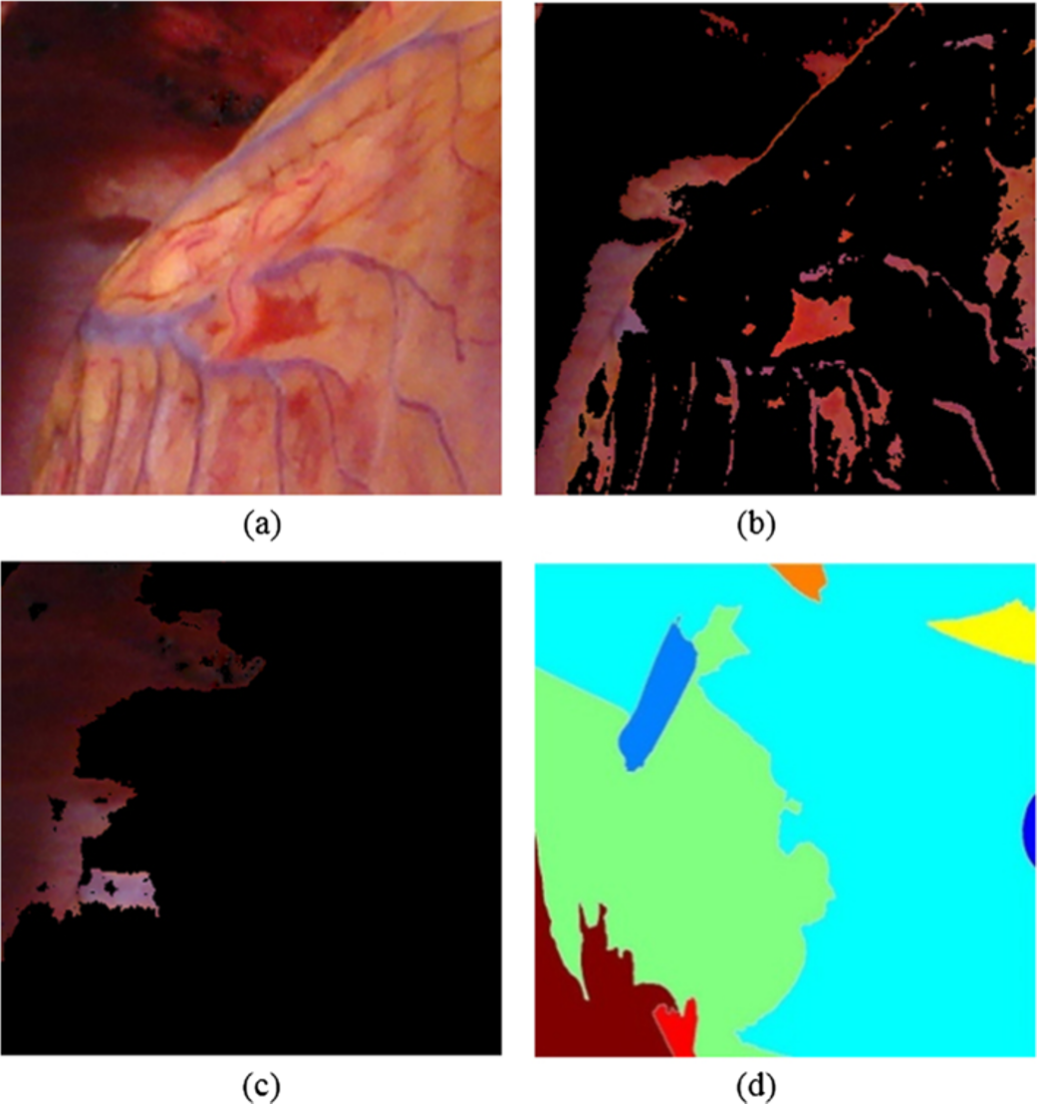
**Vessel segmentation results from some general segmentation algorithms. (a)** Original human heart image. **(b)** Thresholding using Otsu method. **(c)** Region growing method. **(d)** Watershed method.

The thresholding method has been used in the segmentation process for medical images. However, each image has different characteristics from other images, which make the algorithms to choose an optimal threshold a very challenging task. For this reason, any thresholding method may obtain good results for some images while it does not obtain the same result for all images. One of the common threshold methods is Otsu threshold method which applied here as [31], however it work will for homogenous image features as shown in Figure 13 (b).

Similarly, the region growing method is implemented and the segmentation results are found to be not promising. In addition, it is a time consuming method. The result is given in Figure 13 (c). In a like manner, Figure 13 (d) presents the result of the watershed method that is not clear. The classifier methods are normally used for clustering cell like images. However, when used for images of a real human heart it results in incorrect vessel segmentation.

The proposed method goal is to obtain vessels ROI. Several factors make this goal hard to achieve with the current methods. A crucial aspect of segmentation methods is what are best suited to specific applications and type of data. No segmentation method is better than the others are for any purpose. Thus, we have to figure out what available method fits best into vessel ROI in terms of accuracy, speed, and reducing the amount of user interaction. The experimental results obtained in the previous sections could not accurately segment the vessels ROI. As illustrated in Figures 12 and 13 a results of applying different segmentation methods using the human heart images, whereas these segmentation methods are design for a specific purposes and for specific input images.

Furthermore, Figure 12 results shows the ability of medical imaging applications to demonstrate some significant performance advantage (e.g. faster detection) over traditional methods. However, it also result with wrong vessels region detection. In the other hand, Figure 13 shows results of some general segmentation methods in which those methods depends on the input image types, which required different parameters to obtain a better results for different input image. It is unlikely that automated segmentation methods will ever replace experts, but they will likely become crucial elements of medical-image analysis.

**Figure 14:**
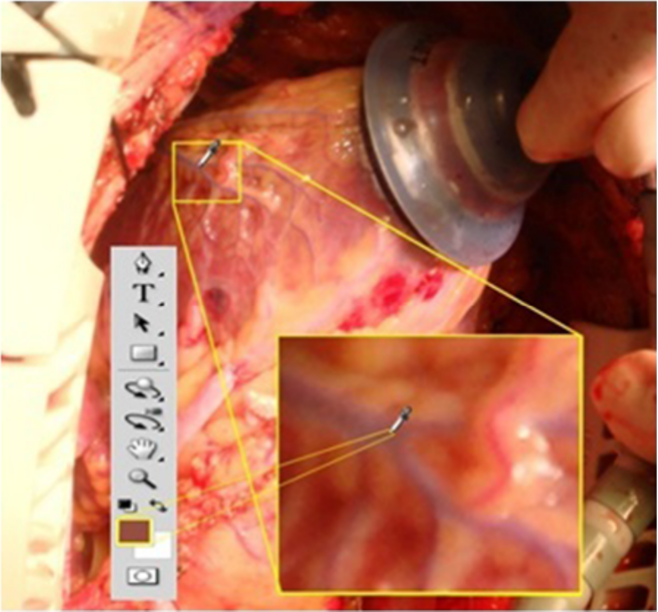
**Human heart surface vessel magnified part of veins using APCS4 Eyedropper tool.**

In some instances, the heart surface fats and tiny epidermis cover the vessels, which result in incorrect vessel segmentation. The transparent properties of the tiny epidermis sometimes also affects the segmentation methods as they are detected as the surface of the heart surface instead of the surface of vessels as shown in Figure 14. The figure shows how the Adobe Photoshop CS4 (APCS4) Eyedropper tool perceives the colour of the vessel, even the vessel look as bluish colour by human perception but the Eyedropper tool detect it as unvessel regions.

Furthermore, the advantage of identifying and segmenting the surface vessels of a human heart from real colour images is it can guide the surgeons to the vessel region in order to perform the surgery and to avoid surgical injuries. In the end, the whole process can be summarised as requiring only a single-view of a real human heart colour image for the segmentation process. It is a semi-automatic algorithm, as user interaction is necessary to obtain a final 3D model of the heart with its 3D vessels. The user is required to click on any part of the vessel regions and the program will run automatically to determine the final segmented vessels of the surface of the human heart.

Finally, the proposed algorithm has a faster segmentation process and ease of use compared to the existing algorithms, i.e. faster compare to other segmentation methods that deal with the same input data, i.e. colour images. In addition, no time required for converting to another colour space. However, the limitation of the proposed vessel segmentation algorithm is that fats and light reflections may cover the whole surface of the vessels in which some vessel regions may not be segmented correctly.

Although there are existing drawbacks due to fats and light reflections, the proposed algorithm shows better performance than existing methods by means of accurate identification and segmentation of the surface vessel of the human heart from a single real colour image. As well, facilitates guiding the surgeons to the vessel ROI with the vessels being visualised over the surface of the heart.

## Conclusion

A heart surface vessel segmentation and reconstruction mechanism is described in this article. This mechanism run in a fast manner for 3D surface vessel reconstruction of a real human heart from a single colour image. Moreover, the reasons for the use of a Decorrelation stretch algorithm are investigated as a main image enhancement process for the vessel ROI where the operation is to be performed. The segmentation process correctly detect the vessels even if fats cover the surface of the heart although it can be affected by the lighting conditions in the colour images.

In addition to the reconstruction process of the segmented vessels, the three axes are needed to view the 3D vessels over the heart model in R3 space. The proposed mechanism employs the information of the pixels in the same manner for the 3D reconstruction process. Furthermore, the mechanism presented here has been justified and validated by conducting tests on hand-labelled ground truth vessel segmentation results of several images of human heart data. Moreover, the Bezier curve approximation technique is used to smooth the result of the 3D vessels segmented model over the 3DHHM. Finally, the results show that the proposed mechanism significantly improves the system performance by achieving superior accuracy rate of up to 94.42%.

## Authors' contributions

All authors have made substantial contributions to conception and design, or acquisition of data, or analysis and interpretation of the data. In addition, authors have been involved in drafting the manuscript or revisiting it critically for important intellectual content and have given final approval of the version to be published. All authors read and approved the final manuscript.

## Authors’ original submitted files for images

Below are the links to the authors’ original submitted files for images. Authors’ original file for figure 1 Authors’ original file for figure 2 Authors’ original file for figure 3 Authors’ original file for figure 4 Authors’ original file for figure 5 Authors’ original file for figure 6 Authors’ original file for figure 7 Authors’ original file for figure 8 Authors’ original file for figure 9 Authors’ original file for figure 10 Authors’ original file for figure 11 Authors’ original file for figure 12 Authors’ original file for figure 13 Authors’ original file for figure 14

## Abbreviations

ROI: Region of interest
3DHHM: Three Dimensional Human Heart Model
MRI: Magnetic resonance imaging
CT: Computed tomography
CTA: Computed tomography angiogram
MRA: Magnetic resonance angiogram
GUI: Graphical user interface
HSI: Hue, saturation, and intensity
HSV: Hue, saturation, and value
APCS4: Adobe Photoshop CS4
